# Prevalence and Assessment of Clinical Management of Sexually Transmitted Infections among Female Sex Workers in Two Cities of India

**DOI:** 10.1155/2011/494769

**Published:** 2011-06-22

**Authors:** A. Das, P. Prabhakar, P. Narayanan, G. Neilsen, T. Wi, S. Kumta, G. Rao, R. Gangakhedkar, A. Risbud

**Affiliations:** ^1^India Office, FHI, H 5 Green Park Extension, New Delhi 110016, India; ^2^Asia Pacific Regional Office, FHI, 19th Floor, Tower 3, Sindhorn Building, 130-132 Witthayu Road, Bangkok 10330, Thailand; ^3^Regional Office for the Western Pacific, World Health Organization, P.O. Box 2932, Manila 1000, Philippines; ^4^Bill & Melinda Gates Foundation, Capital Court Building, 3rd Floor, Left Wing, Olof Palme Marg, Munirka, New Delhi 110 067, India; ^5^National AIDS Research Institute, 73 ‘G' Block, Bhosari, Pune 411026, India

## Abstract

*Objective*. Control of sexually transmitted infections (STIs) among female sex workers (FSWs) is an important strategy to reduce HIV transmission. A study was conducted to determine the prevalence and assess the current clinical management of STIs in India. *Methods*. FSWs attending three clinics for regular checkups or symptoms were screened for study eligibility. A behavioral questionnaire was administered, clinical examination performed, and laboratory samples collected. *Results*. 417 study participants reported a mean number of 4.9 (SD 3.5) commercial clients in the last week. 14.6% reported anal sex in the last three months. Consistent condom use with commercial and regular partners was 70.1% and 17.5%, respectively. The prevalence of gonorrhea was 14.1%, chlamydia 16.1%, and trichomoniasis 31.1% with a third of all infections being asymptomatic. Syphilis seropositivity was 10.1%. *Conclusions*. At study sites, presumptive treatment for gonorrhea, chlamydia, and syphilis screening should continue. Presumptive treatment for trichomoniasis should be considered. Consistent condom use and partner treatment need to be reemphasized.

## 1. Introduction

Globally, sexually transmitted infection (STI) rates are the highest among population subgroups such as sex workers with high rates of partner change and unprotected sex [1–4]. The presence of an STI increases the risk of acquisition and transmission of HIV [5, 6]. Integrated services for sex workers such as peer education, empowerment, condom promotion, and effective treatment for STIs along with structural interventions have demonstrated reductions in STI and HIV prevalence [7–9]. Asymptomatic STIs, more common in females, are usually managed through regular screening and presumptive treatment [10]. Various clinical strategies including one-time and periodic presumptive treatment used in different countries have been evaluated [11] which concluded that presumptive treatment should be included within a package of comprehensive STI services for sex workers. Cost-effectiveness studies among female sex workers (FSWs) in Madagascar [12] and Bangladesh [13] have reiterated the importance and utility of syphilis screening and the presumptive treatment for genital tract infections.

Several STI prevalence studies using multiple diagnostic methods in community and clinic-based settings among FSWs in India from 2000 to 2009 have shown widely varying rates across different parts of the country. The prevalence of gonorrhea and chlamydia ranged from 0 to 19.1% [14, 15] and from 0.9 to 22.6% [16], respectively, while prevalence of syphilis and trichomoniasis was in the range of 1.2–51% [16, 17] and 2–54.1% [14, 18], respectively. HIV prevalence also ranged from 2.2 to 54% [16, 19] at different locations. 

**Figure 1:**
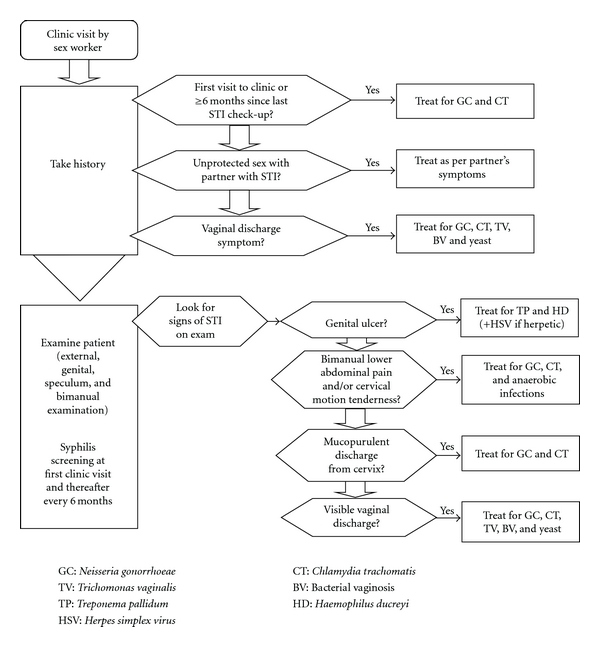
Flowchart for female sex worker visit.

Avahan, the India AIDS initiative of the Bill & Melinda Gates Foundation has been providing HIV prevention services since 2004 to over 200,000 FSWs in 83 districts (of a total of 130 districts) in six high-HIV-prevalence states of India [20]. Avahan works either alongside government or donor-supported nongovernmental organizations (NGOs) or as the sole service provider in a district. The main components of the intervention are peer-led outreach education, condom promotion and distribution, STI clinical services, community empowerment, and structural interventions. The *Clinic Operational Guidelines and Standards* (COGS) [21] developed for Avahan clinics describe the essential STI service package for treating the beneficiaries. The package comprises of syndromic management of symptomatic infections as per Indian national guidelines, presumptive treatment for gonorrhea and chlamydia at the first clinic visit which is repeated if the individual has not attended the clinic for an STI checkup for six months, quarterly clinical STI checkups, and biannual syphilis screening.  Figure 1 shows the Avahan algorithm for clinical management of STIs among FSWs. The STI services were designed at the outset in 2005 based on limited STI prevalence studies among FSWs in India and relevant experience from other countries and modified slightly based on survey data in 2006 [16]. The National STI program adopted the Avahan guidelines for STI control among FSWs in 2007 [22].

The integrated behavioral and biological assessments (IBBAs) are periodic cross-sectional surveys to measure the outcome and impact of Avahan interventions on district-wise community-based samples. Two rounds of IBBAs have been conducted, the first from 2005 through 2007 (two years after the start of Avahan) and the second round concluded recently. The first round in 2005–2007 (IBBA-1) [16], conducted in 29 districts among 25,162 respondents, showed a low prevalence of gonorrhea and chlamydia in most districts. However, some FSWs sites in the cities of Hyderabad and Mumbai had a higher prevalence of cervical infections. A study was conducted at FSW clinics in these cities to determine the prevalence of STIs and assess the current clinical strategies for STI management among clinic attendees. 

## 2. Materials and Methods

From October 2008 to May 2009, 417 consecutive, eligible, and consenting clinic attendees were recruited from three dedicated FSW clinics in two cities of India (Hyderabad and Mumbai). The support for the outreach and clinic services varied across sites.

 The criteria for site selection were the following:

- known high prevalence of gonorrhea and chlamydia based on results from IBBA-1,
- providing clinical services to large numbers of stable (not highly mobile) FSWs as determined from the clinic records,
- operational feasibility for conducting the study defined as proximity to a local laboratory with facilities for conducting required tests and storage of specimens with facilities for transporting specimens to the tertiary laboratory at the National AIDS Research Institute (NARI), Pune, India.

The eligibility criteria for participants were based on rates of partner change (commercial sex at least twice in the last week and/or eight times in the last month) and age between 18 and 40 years. Additionally, those pregnant or under the influence of drugs or alcohol were excluded from the study. 

### 2.1. Study Procedures

At workshops conducted at each site before the initiation of data collection, outreach workers and peer educators of the NGO were oriented about the study. During field visits, they created awareness about the study and encouraged eligible individuals to participate. All FSWs attending the clinic for any reason (STI symptoms, STI checkups, or other reasons) were screened for eligibility criteria. Written or witnessed verbal informed consent was obtained from the individuals found eligible. Trained female investigators from a research agency administered a behavioral questionnaire pertaining to demographics, sexual behavior, condom use, treatment seeking, and past exposure to HIV prevention interventions. 

A detailed clinical history was elicited and clinical examination of the anogenital area including speculum and bimanual examination was carried out by the clinic physician who had been trained on the study protocols. The physicians were trained to elicit signs of cervical infection (cervical discharge, friability, or ectopy) and pelvic inflammatory disease (cervical motion or adnexal tenderness). Findings were recorded in a clinical assessment form. Vaginal swabs for laboratory investigation were collected from all participants during the clinical examination. If endocervical discharge or genital ulcers were detected, endocervical and ulcer swabs were obtained. 

### 2.2. Laboratory Investigations

Saline and potassium hydroxide (KOH) mounts of the vaginal swabs were microscopically examined at the site for *Trichomonas vaginalis *(TV) and *Candida spp.,* respectively. Gram-stained slides of vaginal swabs were scored by Nugent's criteria for bacterial vaginosis (BV), while Gram-stained slides of endocervical swabs were examined for Gram-negative intracellular diplococci and pus cells at the local laboratory. At the tertiary laboratory, infections with *Neisseria gonorrhoeae* (GC) and *Chlamydia trachomatis* (CT) were detected in the vaginal swab samples using transcription-mediated amplification (Gen-Probe APTIMA Combo-2 Assay, Gen-Probe Inc, San Diego, Calif, USA). Vaginal swabs were tested by the nucleic acid amplification technique using polymerase chain reaction method published by Van Der Pol et al. for TV [23].

Sera from all participants were screened for syphilis by rapid plasma reagin (RPR, Span Diagnostics Ltd, India), and confirmation of all RPR-reactive sera was done by *Treponema pallidum* hemagglutination assay (TPHA) using Syphagen TPHA (Biokit, Barcelona, Spain). *Herpes simplex virus* type 2 (HSV-2) IgG ELISA was also performed on all serum samples using EIA-Herpeselect-2 IgG kit (Focus Diagnostics Inc, Cypress, Calif, USA). 

Genital ulcer swabs were tested for *Treponema pallidum *(TP), *Haemophilus ducreyi *(HD), and HSV by multiplex PCR using the method described by Orle et al. [24]. 

### 2.3. Ethical Approval

The study was approved by the ethics committee of NARI, Pune, India and the Protection of Human Subjects Committee of FHI, NC, USA. 

### 2.4. Statistical Analysis

The questionnaire and clinical data of participants were entered into CSPro 3.3 (US Bureau of Census, USA), and univariate and bivariate analyses were performed using SPSS version 17.0 (SPSS Inc, Chicago, Ill, USA). Chi-square tests were used for determining the behavioral and biological correlates of laboratory-confirmed STIs. The performance of the STI flowcharts was done by calculating the sensitivity, specificity, positive, and negative predictive value of the syndromic diagnosis compared to the etiological diagnosis obtained from gold standard laboratory tests for the respective pathogen.

## 3. Results

A total of 517 individuals were approached at the clinics for study participation, of them 468 were found to be eligible. 455 individuals gave informed consent, and 417 individuals completed all study procedures. 

### 3.1. Sociodemographic Profile

The median age of the participants was 30 years, and 80% were unable to read or write. Most of them (96%) had been or were currently married with 70% currently having regular partner/s. Most of the FSWs were street-based (70%) or home-based (21%). The median duration of sex work was three years, and 60% said that they had an additional source of income. 

### 3.2. Risk Behavior

The mean numbers of commercial clients on the last working day and last week were 1.7 and 4.9 (Standard Deviations: 0.9 and 3.5), respectively. While 82.7% said that they had used condoms at their last commercial sexual encounter, 70.1% claimed to be consistently using condoms with commercial partners. Most participants (70.4%) said that they had one or more (mean 1.3, SD 0.7) regular, noncommercial partner/s. Reported condom use with regular partner/s was low, with 29% reporting using a condom at last sex and only 17.5% reporting consistent condom use. A small (14.6%) but not insignificant number of participants reported anal sex in the last three months, and condom use at the last anal sex was 75.4%.

The majority (77.7%) of participants reported an STI or reproductive tract infection (RTI) symptom in the last six months, of whom about two-thirds sought treatment from a trained provider. When asked about the reason for the current clinic visit, 54.5% said that they had been requested by the outreach staff; 28% cited STI/RTI-related complaints, 13% had come for an STI checkup while 4% stated non-STI/RTI symptoms.

### 3.3. Clinical Findings

The majority (74.6%) of participants had clinical symptoms and/or signs of STIs, with 311 of a total of 417 participants being syndromically diagnosed with an STI. The most common syndromes were vaginal discharge (78.5%) and lower abdominal pain (18.7%).Only 12 participants had genital ulcer disease syndrome. 

### 3.4. Laboratory Test Results

Table 1 shows the prevalence of STIs among the study participants. A total of 109 participants (26.1%) had gonorrhea and/or chlamydia; of these 68% presented with symptoms of vaginal discharge and/or lower abdominal pain, while the rest were asymptomatic. Of those with laboratory-confirmed GC/CT, about half (45%) had classical signs such as cervical discharge or friability or ectopy, cervical motion, or adnexal tenderness. The significant clinical correlates associated with GC/CT infection were the presence of abnormal vaginal discharge on speculum examination (*P* = .009) and cervical friability (*P* = .013). More than half of the GC/CT infections were among those relatively new to sex work (<2 years) while chlamydial infection was significantly associated with attending the clinic for the first time (*P* = .023). There was no significant difference in prevalence of STIs (GC, CT, TV, syphilis, and HSV-2 serology) between street-based and home-based sex workers. Of the individuals with laboratory-confirmed trichomoniasis and bacterial vaginosis, 36% and 35%, respectively, did not complain of vaginal discharge.

The performance of the flowcharts for vaginal discharge syndrome against gold standard laboratory tests is shown in Table 2. Sensitivity values for GC and CT were reasonably high while the positive predictive value (PPV) increased when the flowchart was used for detection of cervical infections due to GC and/or CT. 

Table 3 shows the incremental effect of diagnosis of cervical infections by history alone, by history and clinical examination including speculum findings, and by history, clinical examination, and simple onsite laboratory tests. The addition of speculum examination findings significantly improved the sensitivity.

Of the 12 participants who had genital ulcers, multiplex PCR of ulcer swabs showed TP in six, and one had HSV. Of the six individuals with TP, only one had a positive syphilis serology (RPR titer 1 : 2, TPHA-positive). 

## 4. Discussion

The clinic-based study made attempts to recruit participants from the general population of sex workers by awareness-building through the outreach staff. The three clinics selected provide services for regular checkups and general health complaints in addition to STI symptoms. However, a majority of the study participants had STI/RTI-related symptoms and signs. One in every four sex workers attending STI clinic services had laboratory-confirmed cervical infections; additionally, there was a high prevalence of vaginal infections including BV and trichomoniasis. There was no variability in STI prevalence between street-based and home-based sex workers. Presumptive treatment for GC and CT is justified at the study sites given the high prevalence (26.1%) of cervical infections of which a third were asymptomatic. Presumptive treatment with metronidazole for TV, as recommended in other studies [25] should also be considered in this group given the high prevalence (31.1%) with a third of infections being asymptomatic. 

One in every four participants who presented complaining of vaginal discharge had laboratory-confirmed cervical infections. Our study reconfirms that FSWs (all of whom will have a positive risk assessment) with vaginal discharge syndrome should be treated for both cervical and vaginal infections. Additionally, speculum examination detected signs of cervical infection such as abnormal vaginal discharge and cervical friability even in the absence of complaints of vaginal discharge. The clinical diagnosis shows markedly increased sensitivity for predicting cervical infections when speculum examination is added to history-taking. All clinics providing STI services to FSWs should have facilities for speculum examination to improve the diagnosis of gonorrhea and chlamydia.

The prevalence of latent syphilis (RPR of any titer confirmed with TPHA) was 10.1% while high-titer syphilis (RPR titer ≥1 : 8) was 5.8%. The high prevalence of latent syphilis indicates the necessity of providing periodic syphilis screening and appropriate treatment when indicated. Over three-fourths of FSWs were found having anti-HSV-2 IgG antibody. However, only 12 participants were clinically diagnosed with genital ulcer disease (GUD). The ulcer swab showed that the etiology of GUD was due to syphilis in most cases, unlike an earlier study from India which showed HSV and chancroid as the leading causes [26]. 

In addition to the clinical interventions, health education and counseling sessions at the clinics need to reemphasize risk reduction measures including consistent condom use. Since reported condom use with regular partners was very low, renewed efforts should be made to treat the regular partners of FSWs with STIs. Partner treatment strategies should be customized for the local situation. Health care providers need to ask about anal sex and provide appropriate management. 

Reported condom use and STI prevalence in our clinic-based study were compared with the data from IBBA-1 [16] conducted among district-wise community-based samples (using time-location or respondent-driven sampling) of FSWs in 23 districts of the four southern states in 2005–2007. The IBBA showed that self-reported consistent condom use with occasional clients ranged from 36% (Chittoor, Andhra Pradesh, India) to 93% (Kolhapur, Maharashtra, India) and with regular nonpaying partners from 1% (Chittoor) to 58% (brothel-based FSWs, Thane, Maharashtra, India). Consistent condom use with occasional clients and regular nonpaying partners in Hyderabad was 56% and 4%, in Mumbai 73% and 12%, respectively, as reported in the IBBA. In our study, consistent condom use appears to be slightly higher at 70% and 17.5% with commercial and regular partners, respectively.

The IBBA-1 reported overall low gonorrhea prevalence (<5%) from 20 districts while chlamydia prevalence was less than 5% in 15 districts of a total of 23 districts. The prevalence of gonorrhea ranged from 0.2% (Prakasam, Andhra Pradesh, India) to 9.3% (brothel-based FSWs, Mumbai, Maharashtra, India) while chlamydia prevalence ranged from 0.9% (Madurai, Tamil Nadu, India) to 14.2% (street-based FSWs, Thane, Maharashtra, India). In Hyderabad, prevalence of gonorrhea and chlamydia was 6.4% and 6.5%, respectively, while among brothel-based Mumbai FSWs prevalence was 9.3% and 8.5%, respectively as reported in the IBBA-1. The IBBA Round 2 (2009-2010) results have shown a declining trend of gonorrhea and chlamydia in most districts [27]. As mentioned earlier, the sites for our study were selected based on the high prevalence of gonorrhea and chlamydia shown in the IBBA-1. However, our study showed a higher prevalence of gonorrhea and chlamydia probably owing to being a clinic-based sample as compared to the community-based sample in the IBBA. The data illustrate the heterogeneity of India, both in terms of STI prevalence and response, and the challenges of making generalized recommendations for STI control in such a heterogeneous, large country based on findings from two cities. 

Syphilis prevalence in the IBBA-1 ranged from 4.7% (street-based FSWs, Thane, Maharashtra, India) to 51% (Yavatmal, Maharashtra, India), and syphilis prevalence among Hyderabad and brothel-based Mumbai FSWs was 17.4% and 13%, respectively. Syphilis prevalence in our study was lower and may be the result of the intensified efforts by Avahan-implementing agencies for universal syphilis screening (including introduction of rapid, point-of-care syphilis tests) and appropriate treatment. The declining syphilis trends have been confirmed in IBBA-2 [27]. 

Studies from certain sites in medium HIV prevalence states in India have also shown a high prevalence of STIs among FSWs. A study in the year 2000 from a red-light area in Surat, Gujarat [28], India showed prevalence of syphilis 22.7%, trichomoniasis 14.4%, gonorrhea 16.9%, and chlamydia 8.5%. The study also found poor performance of the STI syndrome algorithms against laboratory diagnosis of STIs and recommended exploring additional strategies including presumptive treatment for STI control among FSWs. 'A more recent study from Goa, India by Shahmanesh et al. [29] reported prevalence of trichomoniasis 9.4%, gonorrhea 8.9%, chlamydia 7.3%, and positive-HSV-2 serology 57.2%.

Our study results reveal a fair proportion of asymptomatic cervical infections and minimal correlation with known behavioral or biological factors. Several studies [30–33] have been conducted among sex workers to generate and test algorithms for diagnosing cervical infections through associations with sociodemographic, behavioral, clinical, and biological factors. These studies have also found limited correlations with factors such as duration of sex work, number of commercial clients, classical signs of cervical infections, training and experience of clinicians and onsite laboratory tests (e.g., Gram stain). The general recommendations were to initiate or continue periodic presumptive treatment and the need for rapid, reliable diagnostics for onsite use. 

A technical consultation on presumptive treatment for STIs in 2005 [11] conducted by the World Health Organization, London School of Hygiene and Tropical Medicine, and the Population Council recommended that presumptive treatment should be seen as a way of quickly reducing STI prevalence while other preventive and curative services are being established. In the Avahan program, a package of STI clinical services to address both symptomatic and asymptomatic infections was offered, and overall efforts are ongoing to increase accessibility to STI services by promotion through outreach and provision of services at suitable locations and timings. Routine monitoring data shows that of the FSWs ever contacted through outreach services by December 2008, 86% had visited the project STI clinics at least once while about 25% were availing clinical services each month [34].

This study was a clinic-based study conducted at known high STI prevalence sites in urban areas; hence, the STI prevalence cannot be generalized to other sex work sites in India. The study is not representative of all Avahan or other FSW sites run by the government program. We used clinic-based sampling supported by awareness-building about the study by outreach workers. While we used a “take-all” strategy for all sex worker clinic attendees who met the eligibility criteria, most of the study participants (74.6%) were clinically diagnosed with at least one STI. However, the high prevalence of curable bacterial STIs and RTIs in the study population is a cause for concern. In addition to STIs, earlier studies from India [35] and elsewhere [36] have shown that the presence of an RTI such as BV is also significantly associated with acquisition of HIV infection. The study results indicate the need for national-level periodic STI surveillance to identify the changing patterns and etiologies and intensified efforts for STI control at high prevalence sites for effective HIV prevention. 

## 5. Conclusions

At the study sites, presumptive treatment for gonorrhea, chlamydia, and syphilis screening is justified. Presumptive treatment for trichomoniasis should be considered. FSWs with vaginal discharge syndrome should be treated for both cervical and vaginal infections. All clinics providing STI services for sex workers should have facilities for speculum examination to improve the diagnosis of cervical infections. Consistent condom use and partner treatment need to be re-emphasized. The epidemiological diversity in India indicates the need for periodic surveillance to identify changing patterns and etiologies and intensified efforts for STI control at high prevalence sites. 

## Figures and Tables

**Table 1:** STI prevalence.

Sample	Test	Pathogen	No. Tested	No. of positives with symptoms (%)	No. of positives without symptoms (%)	No. of positives (total)	Prevalence % (95% CI)
Vaginal swab	Gen-Probe APTIMA Combo II	*Neisseria gonorrhoeae* (GC)	417	39 (66.1)	20 (33.9)	59	14.1 (10.8–17.5)
		*Chlamydia trachomatis* (CT)	417	46 (78.7)	21 (31.3)	67	16.1 (12.5–19.6)
		GC and/or CT	417	74 (67.9)	35 (32.1)	109	26.1 (21.9–30.4)

Vaginal swab	Gram stain Nugent's criteria	Bacterial vaginosis	396	182 (64.8)	99 (35.2)	281	71.0 (66.5–75.5)
	PCR	*Trichomonas vaginalis*	399	80 (64.5)	44 (35.5)	124	31.1 (26.5–35.6)
	KOH mount	*Candida spp.*	402	62 (72.9)	23 (27.1)	85	21.1 (17.3–25.5)

Ulcer swab	mPCR	*Treponema pallidum*	12			6	
		*Haemophilus ducreyi*	12			0	
		*Herpes Simplex Virus* (HSV)	12			1	

Serum	RPR + TPHA	Syphilis serology	416			42	10.1 (7.4–13.4)
		High-titer syphilis (≥1 : 8)	416			24	5.8 (3.7–8.5)
	IgG serology	HSV-2	400			307	76.8 (72.3–80.8)

**Table 2:** Performance of FSWs' vaginal discharge flowchart against gold standard laboratory tests.

Pathogen	No. of infected based on laboratory tests	No. of correctly diagnosed by flowchart	Sensitivity (%)	Specificity (%)	Positive predictive value (%)	Negative predictive value (%)
*Neisseria gonorrhoeae*	59	40	67.8	43.0	16.4	89.0
*Chlamydia trachomatis*	67	46	68.7	43.4	18.9	87.9
Cervical infections (GC/CT)	109	76	69.7	45.5	31.2	80.9
Bacterial vaginosis	281	168	59.8	35.7	69.4	26.6
*Trichomonas vaginalis*	124	78	62.9	43.6	33.5	72.3
*Candida spp.*	85	62	72.9	42.6	25.4	85.4
Vaginal infections (BV/TV/Can)	308	188	61.0	41.9	81.4	20.5

**Table 3:** Diagnosis of cervical infections.

	Sensitivity (%)	Specificity (%)	PPV (%)	NPV (%)
*History of vaginal discharge*	67.9	36.9	27.2	76.8
*History + speculum-abnormal vaginal discharge, cervical discharge/friability/ectopy*, *and cervical motion/adnexal tenderness *	83.5	21.4	27.3	78.6
*History + speculum + simple laboratory tests: Presence of Gram −ve intracellular diplococci, >10 pus cells per HPF in endocervical discharge*	85.3	18.8	27.1	78.4
